# Convergent evolution of RFX transcription factors and ciliary genes predated the origin of metazoans

**DOI:** 10.1186/1471-2148-10-130

**Published:** 2010-05-04

**Authors:** Jeffrey SC Chu, David L Baillie, Nansheng Chen

**Affiliations:** 1 Department of Molecular Biology and Biochemistry, Simon Fraser University, Burnaby, British Columbia, V5A 1S6, Canada

## Abstract

**Background:**

Intraflagellar transport (IFT) genes, which are critical for the development and function of cilia and flagella in metazoans, are tightly regulated by the Regulatory Factor X (RFX) transcription factors (TFs). However, how and when their evolutionary relationship was established remains unknown.

**Results:**

We have identified evidence suggesting that RFX TFs and IFT genes evolved independently and their evolution converged before the first appearance of metazoans. Both ciliary genes and RFX TFs exist in all metazoans as well as some unicellular eukaryotes. However, while RFX TFs and IFT genes are found simultaneously in all sequenced metazoan genomes, RFX TFs do not co-exist with IFT genes in most pre-metazoans and thus do not regulate them in these organisms. For example, neither the budding yeast nor the fission yeast possesses cilia although both have well-defined RFX TFs. Conversely, most unicellular eukaryotes, including the green alga *Chlamydomonas reinhardtii*, have typical cilia and well conserved IFT genes but lack RFX TFs. Outside of metazoans, RFX TFs and IFT genes co-exist only in choanoflagellates including *M. brevicollis*, and only one fungus *Allomyces macrogynus *of the 51 sequenced fungus genomes. *M. brevicollis *has two putative RFX genes and a full complement of ciliary genes.

**Conclusions:**

The evolution of RFX TFs and IFT genes were independent in pre-metazoans. We propose that their convergence in evolution, or the acquired transcriptional regulation of IFT genes by RFX TFs, played a pivotal role in the establishment of metazoan.

## Background

All metazoans and many unicellular eukaryotes have functional cilia (also known as flagella) [1]. Both motile and immotile cilia (also known as sensory or primary cilia) hold many receptors for sensing environmental signals. Cilia may offer competitive advantages to ciliated organisms by allowing them to avoid predation and also to track nutritionally rich resources [2]. It is thus not surprising that cilia and most ciliary genes are deeply conserved, both in structure and function, in the "tree of life". Such high levels of conservation suggest a common evolutionary origin [1]. Ciliary defects have been associated with defective development in the nematode *Caenorhabditis elegans *[3] as well as a growing list of devastating human genetic disease conditions collectively called ciliopathies, including polycystic kidney disease (PKD), Bardet-Biedl syndrome (BBS), Alstrome syndrome, Joubert syndrome, Meckel-Gruber syndrome, and primary ciliary dyskinesia [4,5]. In mammals, cilia are found on essentially all cell types, highlighting the critical role cilia play [6]. One essential cellular process in cilia is the intraflagellar transport (IFT) that is responsible for the assembly and maintenance of eukaryotic cilia. The IFT machinery consists of four basic molecular modules: (a) motors, (b) Complex A, (c) Complex B, and (d) BBS complex [7,8].

How IFT genes are regulated at the transcriptional level remained largely unknown until this century when Swoboda and colleagues discovered in *C. elegans *that many IFT genes are regulated by DAF-19, a RFX type transcription factor [3]. Mutations in *daf-19 *resulted in defects in cilia development and constitutive dauer formation [3]. DAF-19 binds to X-box motif, which is a highly conserved *cis*-regulatory element first discovered in mammals [3,9]. Ciliary genes in *C. elegans *often contain one or more putative X-box motifs 100 bp - 250 bp upstream of the coding sequences [3,4,10,11]. In addition, ciliary genes and cilia development in the fruit fly *Drosophila melanogaster *were also suggested to be regulated by RFX TFs [12]. Two RFX genes dRFX[13] and dRFX2 [14] have been identified in *D. melanogaster*. dRFX was identified through a homology search for the RFX DNA binding domain (DBD) and dRFX2 was identified through yeast-one-hybrid (Y1H) screening for transcription factors that bind to a putative promoter sequence [13,14]. Notably, dRFX2 has not been found in the *D. melanogaster *genome sequences, suggesting that it is likely located within the heterochromatin regions (William Gelbart, *personal communication*).

RFX TFs were first identified in mammals as binding proteins of the X-box motif [15]. Through bioinformatics searches and molecular characterization, seven RFX genes--RFX1-7 have been found in mammals [16,17]. Different mammalian RFX genes show differential but overlapping expression patterns [16], suggesting that they have complementary and cooperative roles in regulating genes in many different biological pathways. Indeed, mammalian RFX TFs have been shown to interact with each other and with many additional co-factors [16]. Accumulating evidence confirms that RFX genes regulate development and function of cilia in mammals as well. For instance, RFX3 knockout in mice led to abnormal cilia development in both brain [18] and pancreas [19].

Outside of metazoans, however, there is no evidence suggesting that IFT genes are regulated by RFX TFs. No RFX TFs have been reported in the green alga *Chlamydomonas reinhardtii*, a popular model organism for studying cilia biology. Conversely, RFX TFs exist in organisms including the budding yeast *Saccharomyces cerevisiae *and the fission yeast *Schizosaccharomyces pombe *that do not have cilia [17], suggesting that RFX TFs do not regulate ciliary genes in these organisms. Based on these observations, we hypothesize that IFT genes and RFX TFs evolved independently and that their evolution converged at some point. To test this hypothesis, we have identified and examined IFT genes and RFX TFs in hundreds of fully sequence genomes that have become available recently.

## Results

### Molecular evolution of ciliary genes

**Figure 1:**
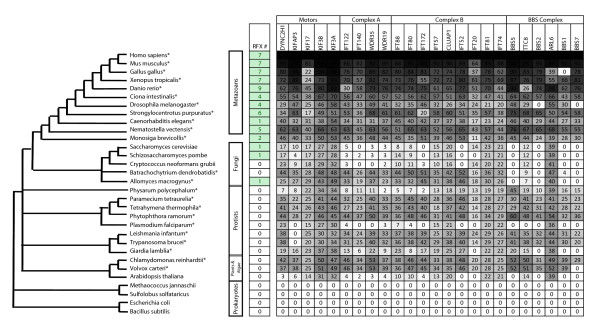
**The conservation of RFX TFs and ciliary IFT components in selected species**. These species were selected to provide a wide sampling of the "tree of life". The phylogenetic relationship between each species was derived from the "Tree of Life Web Project" [44]. Species indicated with "*" have ciliated cells based on published evidence. The 'RFX #' column shows the number of putative RFX TFs identified in this project or reported previously. The grey scale table shows the sequence conservation of individual ciliary components in each species. Darker shade represents higher sequence similarity and conservation. The numbers in each box indicate the percent identity revealed by the alignments between IFT genes and their corresponding human orthologs.

Cilia have been observed to exist in many organisms including mammals, fruit flies, and *C. elegans*. Here, we examine the conservation of cilia by examining the ciliary components identified through searches for human orthologs. In total, we have examined the sequenced genomes of 153 species ranging from metazoans to fungi and plants. The ciliary components examined here include: (1) Five genes from the Motor module (DYNC2H1, K1FAP3, KIF17, KIF3B, and KIF3A); (2) Four from the Complex A module (IFT122, IFT140, WDR35, and WDR19); (3) Nine from the Complex B module (IFT88, IFT80, IFT172, IFT57, CLUAP1, IFT52, IFT20, IFT81, and IFT74); (4) Six from the BBS complex (BBS5, TTC8, BBS2, ARL6, BBS1, and BBS7) [7,8] (Figure 1). We present results from 31 representative species in Figure 1. Most of the ciliary genes examined are strongly conserved in all metazoans ranging from the sea anemone (*Nematostella vectensis*) to human (*Homo sapiens*) (Figure 1). The unicellular choanoflagellate *Monosiga brevicollis*, which have been regarded as the closest extant relative of the last unicellular ancestor of metazoans [20], also have well conserved ciliary genes. Many ciliated protists, including *Paramecium tetraurelia*, *Tetrahymena thermophila*, and *Phytophthora ramorum *have most of the ciliary genes, consistent with previous reports [21]. Also in agreement with previous reports [22,23], we have identified conserved ciliary genes in the unicellular algae *Chlamydomonas reinhardtii *and its closely related multicellular organism *Volvox carteri*. Protists *Giardia lamblia *and *Physarum polycephalum *have ciliary features that are similar to cilia development in mammals [24-27]. However, we observe reduced similarity for all ciliary genes in these two species, suggesting that these ciliary genes in protozoa are fast evolving [28]. The apicomplexan parasite *Plasmodium falciparum *lacks many ciliary genes, consistent to the idea that the apicomplexan parasites may have an entirely different ciliary assembly mechanism [29]. Among the 51 sequenced fungi, we found only two species, *Allomyces macrogynus *and *Batrachochytrium dendrobatidis*, have conserved IFT genes (Figure 1). Interestingly, both species lack most components of the BBS complex. These observations are consistent with previous proposal that cilia were lost independently in many fungal species in evolution [30,31]. Taken together, our comparative identification and analysis of IFT genes suggest that IFT genes are deeply conserved and can be found in all metazoans, most unicellular eukaryotes, and some fungi, but they do not exist in plants such as *Arabidopsis thaliana *and prokaryotes [1,21] (Figure 1).

### Molecular evolution of RFX TFs

**Figure 2:**
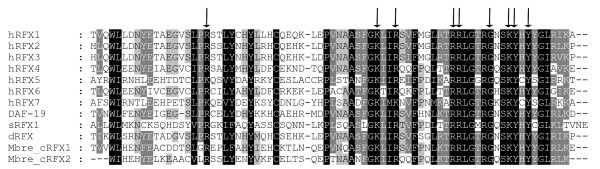
**DBDs of RFX TFs are highly conserved**. Representative DBDs from humans (hRFX1-7), *C. elegans *(DAF-19), *D. melanogaster *(dRFX), *S. serevisiae *(sRFX1), and *M. brevicollis *(Mbre_cRFX1 and Mbre_cRFX2). DBDs from different species show high similarity at the peptide level. Nine residues of DBD that directly contact DNA (indicated by arrows) are essentially identical for all RFX TFs.

Using well defined RFX DBD peptide sequences (76 amino acids long) (Figure 2) from human [16], *C. elegans *[3], *D. Melanogaster *[13], and *S. Cerevisae *[32] as queries, we searched the genomes of the same 153 species for RFX TFs. Because the known RFX DBDs in yeast as well as humans show very high similarity, we used very stringent criteria to look for new RFX TFs. We only consider proteins whose putative RFX DBD show at least 40% percentage identity (PID) to the queries (see Methods). RFX DBD has been shown to contain nine residues that have direct contact with DNA sequences (X-box motifs) [33]. All nine residues are highly conserved in all known RFX DBDs (Figure 2). Therefore we also required that the DBDs of candidate RFX TFs contain all of these nine conserved residues.

We found candidate RFX TFs in all sequenced metazoan genomes (Figure 1). In addition to the RFX TFs that have been reported previously, including seven RFX TFs found in mammals [16], DAF-19 in *C. elegans *[3], and dRFX [13], we found many RFX genes that have not been described previously. We have identified seven RFX genes (RFX1-7) in all vertebrate genomes except fish genomes, which have nine putative RFX genes (RFX1-9). We have also identified four RFX genes in *Ciona intesttinalis*, six in the purple sea urchin (*Strongylocentrotus purpuratus*), and five in the sea anemone (*Nematastella vectensis*). In *D. melanogaster*, in addition to the two RFX genes reported previously--dRFX and dRFX2, we have identified a novel RFX TF, which we named *dRFX1*. Interestingly, among all metazoans examined, nematodes including *C. elegans *are the only organisms that possess just one RFX gene.

RFX TFs are also found in some non-metazoans. Of the 51 fungus species examined, we identified single RFX TFs in 44 species, including the budding yeast *Saccharomyces cerevisiae *and the fission yeast *Schizosaccharomyces pombe*, as previously reported [17], as well as a ciliated fungus *Allomyces macrogynus*, whose genome was recently sequenced by the Fungal Genome Initiative of the Broad Institute http://www.broadinstitute.org/annotation/fungi/fgi/. All unicellular organisms we have examined possess either one RFX gene (fungi) or none except for the choanoflagellates. For example, *M. brevicollis*, which was recently sequenced [20], contain two genes (Mbre_cRFX1 and Mbre_cRFX2) with well-defined RFX DBDs.

RFX DBD sequences are the defining features of all known RFXs and show high similarity (>40% PID) to each other. However, there are a small number of additional proteins that contain domains that show weaker similarity (<30% PID) to known RFX DBDs. In particular, a gene (ARID2) in the human genome contains a RFX-like domain that shows 29% PID to the human RFX1 DBD. Among the nine residues that have direct contact with DNA sequences, five can be found in the RFX-like domain found in ARID2. ARID2, whose function as a transcription factor has not been well studied, has orthologs in all mammals as well as other vertebrates (data not shown). Additionally, a gene in *M. brevicollis *also shows weak similarity (27%) to known RFX DBDs (five of the nine residues that have direct contact with DNA are conserved). We name this novel gene Mbre_cRFX3. Because of their low similarity to known RFX DBDs, these RFX like genes--ARID2 and Mbre_cRFX3--are not regarded as RFX TFs in this project and thus are not examined further. No RFX genes have been found in any bacteria, ancient bacteria, or plants (Figure 1).

**Figure 3:**
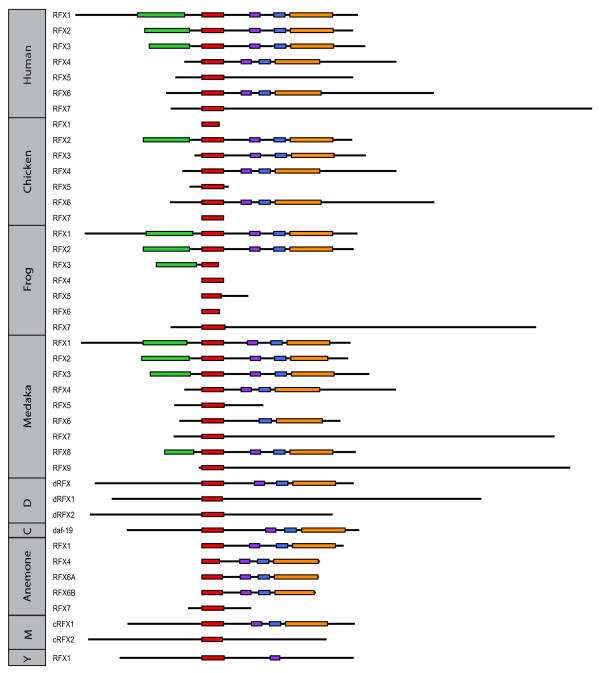
**Predicted protein domains of RFX TFs in representative species**. The defining domain of all RFX TFs--DBD--is shown in red. Other domains including the activation domain (green), the B domain (purple), C domain (blue), and D domain (orange) are not present in all RFX TFs. In the left column, Y stands for the budding yeast *S. cerevisiae*, M for *Monosiga brevicollis*, C for *C. elegans*, and D for *D. melanogaster*.

**Figure 4:**
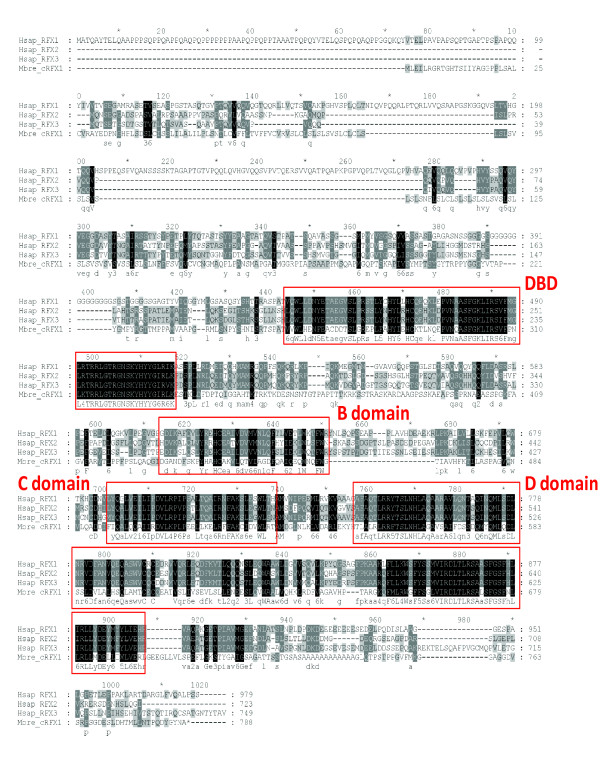
**Sequence alignment between *M. brevicollis *Mbre_cRFX1 and Human RFX1-3 with all functional domains highlighted**. Amino acid residues are color coded with darker color representing higher conservation. Putative functional domains are encircled and labeled.

DBDs in the two putative RFX TFs in *M. brevicollis *are essentially indistinguishable from the DBDs in previously characterized RFX TFs with ~70% PID at the peptide level. All nine residues that make direct contacts with X-box motifs are conserved [33] (Figure 2, residues indicated with arrows). In addition to the DBDs, Mbre_cRFX1 also shares other functional domains within known RFX TFs including the dimerization domains (DD), and the extended dimerization domains (B and C domains), which exist in all mammalian RFX TFs except RFX5 and RFX7 [16,17] (Figure 3). Aligning Mbre_cRFX1 to human RFX1-3 shows clear alignment for conserved DBD, DD, and extended dimerization domains (B and C domains) (Figure 4). None of the *M. brevicollis *RFX TFs have readily identifiable activation domains (AD). The lack of typical AD in RFX TFs in *M. brevicollis*, *C. elegans*, *D. melanogaster*, and sea anemone (Figure 3) suggests that AD might have been acquired later in metazoan evolution. Alternatively, their ADs have yet to be identified and characterized. Mbre_cRFX2 has a readily identifiable DBD but lacks other conserved domains, which is similar to the human RFX5 and RFX7 that lack other domains (Figure 3). The presence of DBD (in both Mbre_cRFX1 and Mbre_cRFX2) and other conserved protein domains (in Mbre_cRFX1) suggest that they may function in transcriptional regulation of gene expression in *M. brevicollis*. However, their target genes remain to be identified.

**Figure 5:**
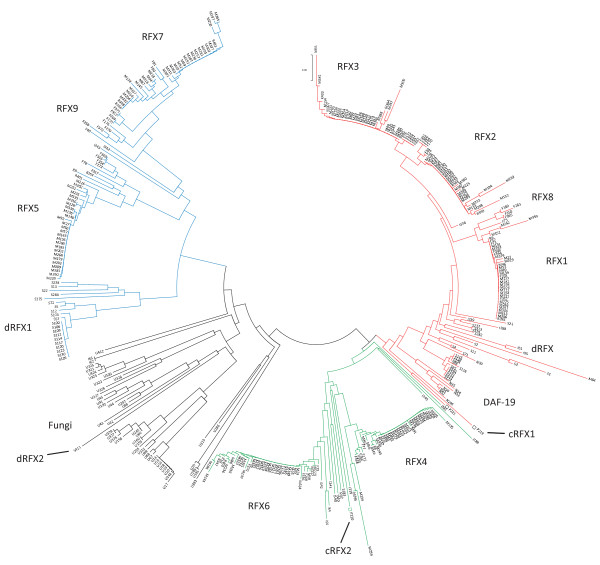
**The phylogenetic tree of all RFX DBDs found in this study**. Each distinct group of RFX is labeled. Labels for each putative RFX refer to records in Additional file 1. The colored branches indicate three major groups of RFX TFs: RFX1-3 in red, RFX4-6 in green, and RFX5-7 in blue. The fish RFX8 TFs cluster with the RFX1-3 group, while the fish RFX9 TFs cluster with the RFX5-7 group. All nematodes are grouped together (labeled DAF-19) with the RFX1-3 group. Some insects RFX TFs (labeled as dRFX) group with RFX1-3, while others (labeled dRFX1) with RFX5-7. *M. brevicollis *(cRFX1 and cRFX2) are shown in open squares (□). *Drosophila *dRFX2 [14] is shown in the tree but it is not found in the sequenced *D. melanogaster *genome nor any sequenced *Drosophila *genome. It is likely located in the heterochromatic region (William Gelbart, *personal communication*). The phylogenetic tree was inferred using the Neighbor-Joining method [45]. Phylogenetic analysis was performed using MEGA4 [46].

To further examine the relationship between the *M. brevicollis *RFX TFs and those identified in mammals and other species, we constructed a phylogenetic tree that contains all known and putative RFX TFs based on the similarity between the DBD domains (Figure 5). Sequences outside of the DBDs are excluded from analysis since they are often very diverse and are not readily alignable. Previous analysis of mammalian RFX TFs revealed three groups: RFX1-3, RFX4-RFX6, and RFX5-RFX7 groups [16], which is generally consistent with this phylogenetic tree with newly identified members (Figure 5). The tree shown in Figure 5 contains an additional clade (shown in black), which contains RFX TFs identified in fungal genomes and, interestingly, *dRFX2 *in *D. melanogaster *[14]. Fungus RFX RFs (members in the Fungus clade) and RFX5-RFX7 TFs show similar domain compositions with all members lacking B, C, and D domains, which are found in the RFX1-3 and RFX4-RFX6 TFs (Figure 3). The inferred phylogenetic tree clearly shows that the Mbre_cRFX1 fits into the RFX1-3 group, while Mbre_cRFX2 fits into the RFX4-6 group. Mbre_cRFX3, which show weaker similarity to known DBDs, clusters closer to DBDs of the RFX5-7 groups. However, as mentioned before, we did not include Mbre_cRFX3 in the phylogenetic tree. The phylogenetic relationship between *M. brevicollis *and previously identified RFX TFs suggest that these three RFX TFs families were established before the split between choanoflagellates and metazoans.

In the inferred phylogenetic tree, the nematodes are the only metazoans that have only one RFX TF--DAF-19, which groups together with the mammalian RFX1-3 group (Figure 5). It was proposed previously that prior to the complete sequencing of the *C. elegans *genome, more RFX TFs should exist in *C. elegans *[17]. However, exhaustive searches of the completed *C. elegans *genome revealed no traces of additional RFX genes, suggesting that RFX genes corresponding to other RFX groups (RFX4-6 and RFX5-7) were lost in the last common ancestor of the nematode species. In fact, none of the seven sequenced nematode genomes have more than one RFX TF (Additional file 1).

### Evolutionary relationship between ciliary genes and RFX TFs

The above comprehensive identification of IFT genes and RFX TFs shows clearly that all metazoans have both ciliary genes and RFX genes. Since IFT genes have been demonstrated to be regulated by RFX TFs in *C. elegans*, *D. melanogaster*, and humans, IFT genes in all metazoans are likely regulated by RFX TFs. Our analysis strongly suggests that IFT genes and RFX TFs evolved independently. In addition to the budding yeast (*S. cerevisiae*) and fission yeast (*S. pombe*), we have identified 41 fungus species that have single RFX genes but no IFT genes, thus RFX genes in these species do not regulate ciliary genes expression. Indeed, Crt1/RFX in the budding yeast plays a role in DNA damage response [34]. Outside of metazoans, only two sequenced genomes have both IFT and RFX genes, the choanoflagellate *M. brevicollis *and the fungus *A. macrogynus*. Outside of metazoans, choanoflagellates, and fungi, none of the sequenced genomes possess a single RFX gene, regardless of the possession of IFT genes.

## Discussion

This is the first project to comprehensively identify and compare RFX TFs in the entire "tree of life" since Emery and colleagues described RFXs in humans (RFX1-5), mice (RFX1-3 and RFX5), *C. elegans*, and the budding and fission yeasts domains more than a decade ago [17]. In this paper, we identified for the first time (1) nine RFX genes in all sequenced fish genomes; (2) two RFX genes in the choanoflagellate *M. brevicollis *genome; (3) single RFX genes in many fungus genomes. Additionally, we have identified RFX genes in many vertebrates. Furthermore, we have identified a third RFX (*dRFX1*) in the fruit fly *D. melanogaster*. Based on our phylogenetic analysis of all RFX TFs identified in the "tree of life", we have confirmed the hypothesis proposed by Emery and colleagues that *C. elegans *has lost RFX genes as it evolved [17].

**Figure 6:**
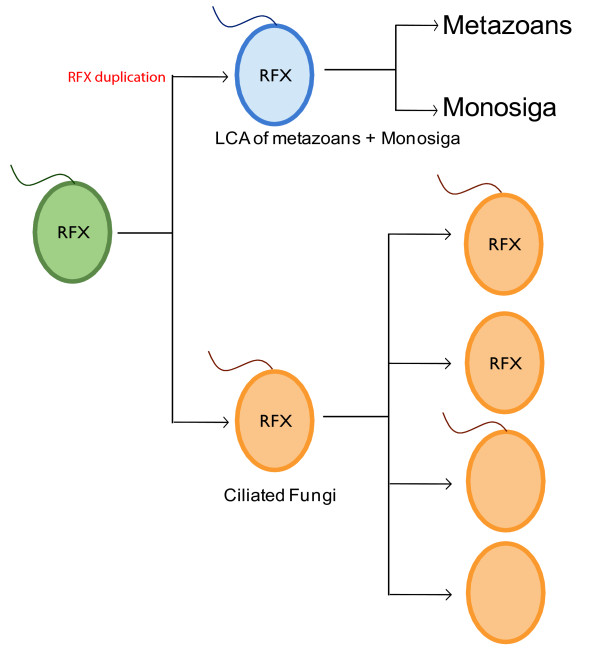
**RFX TF-mediated transcription and the origin of metazoans**. The common ancestor of metazoans, choanoflagellates, and fungi was likely a ciliated unicellular eukaryote with a single RFX TF. Over the course of evolution, some fungi lost RFX TFs while preserving cilia, some lost cilia but kept RFX, and some lost both. Only a few fungi kept both cilia and RFX. The last common ancestor (LCA) of *Monosiga *and metazoans preserved both cilia and RFX.

More importantly, comparative analysis of the molecular evolution of IFT genes and RFX genes revealed a compelling converging relationship between these two gene groups, which is summarized in a model illustrated in Figure 6. We propose that the common ancestor of metazoans, choanoflagellates, and fungi was ciliated and had one RFX gene. Even though the common ancestor of all fungus species was ciliated and had one RFX gene in some fungus species, including *Batrachochytrium dendrobatidis*, inherited cilia but lost RFX, while other species, including budding yeast and fission yeast, lost their cilia but retained RFX, and some species, including *Cryptococcus neoformans grubii*, lost both cilia and RFX TFs, leaving only a few fungal species, including *Allomyces macrogynus*, that retain both RFX TFs and cilia (Figure 1 and Figure 6). In ciliated fungus species, which do not have RFX genes, ciliary genes are likely regulated by factors other than RFX TFs. In contrast, the common ancestor of metazoans and choanoflagellates was ciliated and had multiple RFX genes. The plurality of RFX genes was probably due to gene duplication (Figure 6). The expansion of the RFX gene family, in the common ancestor of metazoans and choanoflagellates, might have provided a platform for the development of interactions between RFX TFs and IFT genes and the establishment of transcriptional regulatory relationships between RFX TFs and IFT genes in metazoans. The convergent molecular evolution of IFT genes and RFX TFs might have provided a pivotal driving force in the emergence and evolution of metazoans.

The evolution of multicellular metazoans from a unicellular protozoan ancestor represents a major and what we consider to be the most spectacular transition in the "history of life". This transition is demonstrated by the abrupt appearance of a huge variety of metazoans in the fossil record approximately 560 million years ago during the Cambrian explosion [35]. Many environmental, ecological, and other evolutionary factors have been proposed to have contributed to this transition [36,37]. Great efforts have been made to understand this transition by studying protein-coding regions of numerous genes and gene families that are ubiquitous in and limited to metazoans. Findings obtained in these studies showed that many genes and gene families previously found to be expressed only in metazoans are also found in choanoflagellates giving evidence that metazoans arose from choanoflagellates. For example, work by King and colleagues clearly demonstrated that choanoflagellates have a receptor tyrosine kinase that is found in metazoans but not in other eukaryotes [35]. Manning and colleagues searched the sequenced choanoflagellates *M. brevicollis *genome [20], and identified a highly elaborate tyrosine kinase signaling network [38]. Many additional genes are shared by *M. brevicollis *and metazoans, including cadherin, which are essential for metazoan development [39], and transcription factors such as P53 and Myc [20]. These findings encouraged additional large scale searches, including the UNICORN (unicellular opisthokont research initiative) project [36], for genes and gene families critical for the transition from unicellularity to multicellularity. However, accumulating evidence is showing that these genes predated the origin of metazoans and played different roles from their counterparts in metazoans. Thus these genes, even though some have been co-opted to perform novel functions in metazoans, are probably not be the main driving force underlying the transition from unicellular protozoans to multicelluar metazoans.

What then was the main factor driving this transition? In contrast to coding sequences of genes, which are usually under strong purifying selection, regulatory sequences show much more rapid evolution. Compelling evidence suggests that changes in *cis*-regulatory sequences and transcriptional regulation in general play a pivotal role in evolution [37,40]. Kingsley and colleagues recently identified changes in *cis*-regulatory modules that dictate dramatic changes in pigmentation in sticklebacks and humans [41]. Thus the transition from unicellular flagellates to multicellular metazoans may have been driven by innovations at the transcriptional level. The convergent evolution of RFX TFs and ciliary genes (IFT genes in particular) in the common ancestor of metazoans and choanoflagellates prompt us to propose that the acquired tight control of ciliary genes at the transcription level by RFX TFs served as one of the critical driving forces in the establishment of multicellularity and the rise of metazoans.

## Conclusion

RFX TFs and IFT genes evolved independently in pre-metazoans and their convergence, or the acquired transcriptional regulation of IFT genes by RFX TFs, may have played a pivotal role in the establishment of metazoan.

## Methods

### Data sources

All sequence data (both genomic DNA sequences and gene annotation data including cDNA and protein sequences) were downloaded from public databases. The list of genomes and the data source are described in Additional file 2. The initial set of DNA binding domains that were used as queries for BLAST searches were taken from Human RFX1-7 [16], *C. elegans *DAF-19 [3], *D. melanogaster *dRFX [13], and yeast RFX1 [32].

### Identification of RFX TFs

We carried out similarity searches using WU-BLAST (version 2.2.6; http://blast.wustl.edu) with e-value 0.01 and without sequence filter (option -F). The initial set of DBDs was used as query to search against all the mammalian proteomes (entire collection of protein peptides). The resulting DBDs were added to the query list and used to search against arthropods. The iteration of adding DBD and blasting continues until all species have been searched. A hit is accepted as a candidate DBD if the corrected percent identity over the entire domain length is >= 40%. The corrected percent identity was calculated as the number of identical positions divided by total length of the query. We also searched for candidate RFX TFs in genome sequences (DNA sequences) to ensure that no RFX TFs have been missed in the gene annotations.

### Identification of ciliary genes

We carried out similarity searches using WU-BLAST (version 2.2.6; http://blast.wustl.edu) with e-value 0.01 and without sequence filter (without -F). Human protein sequences were taken from NCBI and used as queries (See accession number in Additional file 3). PID was calculated as the number of identical amino acids reported by WU-BLAST over the entire length of the query.

### Phylogenetic analysis

Phylogenetic analysis was done using MEGA4 [42]. Multiple sequence alignment was done using CLUSTALW (included in META4) with default settings. Phylogenetic trees were inferred using the Neighbor-Joining method.

### Functional domain identification and analysis

Sequences for activation, B, C, and D domains were taken from previous publications. The multiple sequence alignment was performed for each domain and used as input for hmmbuild to generate a HMM profile for each domain. hmmsearch was used to scan the proteome of selected species to find regions of similar profile. Both hmmbuild and hmmsearch are part of the HMMER suite [43]http://hmmer.janelia.org.

## Authors' contributions

NC and DLB conceived the study. JSCC and NC conducted the experiments and wrote the manuscript. All authors have read and approved the final manuscript.

## Supplementary Material

Additional file 1 **List of all putative RFX genes**. All RFX TF genes identified and shown in Figure 5 are included in the table contained in this file. For each RFX gene, the table provides an ID (shown in the first and second columns) that is used for display in Figure 5, species name, common name of the species, phylum, proposed gene name, chromosomal coordinates, as well as the sequence of the RFX DBD identified in the gene. Click here for file

Additional file 2 **List of genomes processed in this project**. This file lists genomes that are found to contain RFX TF genes. For each genome, the file provides the species name, database from which the genome is downloaded and the version of the database. Click here for file

Additional file 3 **Database information of the IFT genes used as queries**. List of IFT genes and their corresponding NCBI accession numbers. Click here for file
